# Mapping the Effect of Interictal Epileptic Activity Density During Wakefulness on Brain Functioning in Focal Childhood Epilepsies With Centrotemporal Spikes

**DOI:** 10.3389/fneur.2019.01316

**Published:** 2019-12-19

**Authors:** Anna Elisabetta Vaudano, Pietro Avanzini, Gaetano Cantalupo, Melissa Filippini, Andrea Ruggieri, Francesca Talami, Elisa Caramaschi, Patrizia Bergonzini, Aglaia Vignoli, Pierangelo Veggiotti, Azzura Guerra, Giuliana Gessaroli, Margherita Santucci, Maria Paola Canevini, Benedetta Piccolo, Francesco Pisani, Giuseppe Gobbi, Bernardo Dalla Bernardina, Stefano Meletti

**Affiliations:** ^1^Neurology Unit, OCB Hospital, AOU Modena, Modena, Italy; ^2^Department of Biomedical, Metabolic, and Neural Sciences, University of Modena and Reggio Emilia, Modena, Italy; ^3^Italian National Research Council, Parma Research Unit, Parma, Italy; ^4^Child Neuropsychiatry, University of Verona, Verona, Italy; ^5^Child Neuropsychiatry Unit, IRCCS, Institute of Neurological Sciences, Bellaria Hospital, Bologna, Italy; ^6^Physical Medicine and Rehabilitation Unit, Sant'Andrea Hospital, University of Rome La Sapienza, Rome, Italy; ^7^Pediatric Neurology Unit, AOU Modena, Modena, Italy; ^8^Department of Health Sciences, University of Milano, Milan, Italy; ^9^Pediatric Neurology Unit, V. Buzzi Hospital, University of Milan, Milan, Italy; ^10^Child Neuropsychiatry, Department of Medicine and Surgery, University of Parma, Parma, Italy

**Keywords:** CECTS, epileptic discharges frequency, language network, BOLD, cognition, centrotemporal spikes

## Abstract

Childhood epilepsy with centrotemporal spikes (CECTS) is the most common type of “self-limited focal epilepsies.” In its typical presentation, CECTS is a condition reflecting non-lesional cortical hyperexcitability of rolandic regions. The benign evolution of this disorder is challenged by the frequent observation of associated neuropsychological deficits and behavioral impairment. The abundance (or frequency) of interictal centrotemporal spikes (CTS) in CECTS is considered a risk factor for deficits in cognition. Herein, we captured the hemodynamic changes triggered by the CTS density measure (i.e., the number of CTS for time bin) obtained in a cohort of CECTS, studied by means of video electroencephalophy/functional MRI during quite wakefulness. We aim to demonstrate a direct influence of the diurnal CTS frequency on epileptogenic and cognitive networks of children with CECTS. A total number of 8,950 CTS (range between 27 and 801) were recorded in 23 CECTS (21 male), with a mean number of 255 CTS/patient and a mean density of CTS/30 s equal to 10,866 ± 11.46. Two independent general linear model models were created for each patient based on the effect of interest: “individual CTS” in model 1 and “CTS density” in model 2. Hemodynamic correlates of CTS density revealed the involvement of a widespread cortical–subcortical network encompassing the sensory-motor cortex, the Broca's area, the premotor cortex, the thalamus, the putamen, and red nucleus, while in the CTS event-related model, changes were limited to blood–oxygen-level-dependent (BOLD) signal increases in the sensory-motor cortices. A linear relationship was observed between the CTS density hemodynamic changes and both disease duration (positive correlation) and age (negative correlation) within the language network and the bilateral insular cortices. Our results strongly support the critical role of the CTS frequency, even during wakefulness, to interfere with the normal functioning of language brain networks.

## Introduction

Epileptic disorders of childhood and adolescence are challenging conditions, as the repetition of seizures and epileptic discharges (EDs) can have tremendous impact on the developing brain. Childhood epilepsy with centrotemporal spikes (CECTS) is the most common type of “self-limited focal epilepsy” (1), also known as benign epilepsy with centrotemporal spikes (BECTS) or Rolandic epilepsy, representing between 15 and 20% of epilepsies in children between 5 and 14 years of age (2). The prevalence of CECTS is estimated to be ~2% in children, and it is four times more common than typical absence epilepsies (3). It is known to be age dependent, presumably genetic, and mainly occurs at developmentally critical ages. Generally, CECTS is characterized by infrequent focal sensorimotor seizures involving the face during sleep, which may secondarily generalize, reflecting non-lesional cortical excitability from Rolandic and perysilvian regions (4). The prognosis is usually considered to be excellent. Nevertheless, over the past years, some investigators have questioned whether BECTS is indeed benign, considering the variety of different presentations associated with the disorder, thus renamed it CECTS instead of BECTS. It is not uncommon for CECTS to be associated with neuropsychological deficits, especially in visuospatial and verbal fluency tests, language (5) and memory (6) and behavioral problems, such as aggressive behavior, social problems, depression, and attention deficits (7–9). Location of spikes seems to be related to the different selective cognitive deficits in children with CECTS, suggesting an overlap between cortical areas subserving complex cognitive functions and interictal abnormalities sources (10). Different aspects of CECTS were reported to influence cognitive abilities, namely, the age at onset, duration of disease, number of seizures, and antiepileptic drugs (10–12). Nowadays, a causative role of ED is gaining prominence as the predominant mechanism by which epilepsy interferes with the normal organization of oscillatory brain networks, hence causing cognitive deficits (13, 14). A recent electroencephalophy (EEG)-functional MRI (fMRI) study dynamically captured changes in networks' synchronization across different EEG discharge periods in children with CECTS, highlighting the effect of interictal epileptiform activity [represented by centrotemporal spikes (CTS)] “per se” on cognitive functions (15). More than the single ED event, abundance (or frequency) of ED in CECTS is considered a risk factor for epileptogenesis (16) and deficits in cognition processing (17), especially during sleep. Recently, altered widespread functional connectivity patterns were observed in CECTS with spike-wave index during non-rapid eye movement sleep ≥50% compared with the spike-wave index ≤ 50% group, and these alterations were associated with a worse cognitive profile, while no relationship was detected with age of epilepsy onset, disease course, years of education, and total number of seizures (18). For epileptiform activity in wakefulness, it is shown that reading cognitive performances in children with CECTS were higher disrupted when the awake EEG showed high density of spikes than when the EEG was spike free (19). Moreover, previous EEG-fMRI studies documented that ED are associated with the involvement of cortico-subcortical circuits even remote from the seizure onset zone, relevant for the occurrence of the neurodevelopment and neurocognitive impairments (13–15, 20, 21). Despite these premises, the metabolic effect of the ED density measure (i.e., the number of ED for time bin) on the brain function in CECTS has not been explored to date, either in sleep and awake. In this work, we aim to fill this gap by investigating, specifically, the BOLD correlates of the diurnal ED density in a cohort of patients with CECTS and to correlate the revealed hemodynamic patterns with patients' clinical and cognitive measures. We hypothesized that, in case of higher ED frequency, the metabolic counterpart of this quantitative parameter would be able to explain, even partly, the worse clinical and cognitive functioning observed in some CECTS patients by means of the involvement of critical brain hubs and networks.

## Methods

### Study Population

Twenty-seven patients with CECTS [21 male; mean age, 9.7 ± 2.83 years; median age, 9 years (range, 6–17); mean age of epilepsy onset, 7.8 ± 2.6 years; median age, 7 years (range, 2–13)] were selected. Among these, 16 patients age between 7 and 9 years old, 6 patients between 10 and 12 years old, and the remaining 5 patients between 13 and 17 years old. The human ethic committee of the University of Modena and Reggio Emilia approved this study, and written informed consent was obtained from parents and assent from patients. Patients were required to have a clinical diagnosis of CECTS in accordance with the International League Against Epilepsy classification (22) with a history of at least two clinical seizures characterized by simple partial, often facial, and motor or tonic–clonic seizures during sleep and an EEG showing sleep-activated CTS.

The exclusion criteria were (a) any other epilepsy than CECTS, (b) pathological abnormality on conventional MRI, (c) other accompanying neurologic disorders such as cerebral palsy, brain tumor or neurometabolic diseases, and intellectual disability, and (d) head motion while scanning exceeding 3 mm in translation or 3° in rotation.

Before the EEG-fMRI study, within a 15-day window time, the patients were visited by the referring epileptologist and their clinical and EEG features updated. With the exception of two boys (patients 4 and 23) with left-handedness, all patients were right-handed (23).

### EEG-fMRI Protocol

All the recruited patients were scanned in the early afternoon, without sleep deprivation; no sedation was used.

Scalp EEG has been recorded by means of a 32-channel MRI-compatible EEG recording system (Micromed, Mogliano Veneto, Italy). Electrodes were placed according to conventional 10–20 locations. Before in-magnet EEG recording, 10 min of out-of-magnet EEG was collected in a room beside the scanner. Foam pads were used to help secure the EEG leads, minimize motion, and improve patient comfort. Data were transmitted via an optic fiber cable from the amplifier (1,024 Hz sampling rate) to a computer located outside the scanner room. To avoid saturation, the EEG amplifiers have a resolution of 22 bits with a range of ± 25.6 mV.

Patients' behavior has been constantly observed and recorded by means of a small camcorder positioned on the head coil inside the scanner pointing to the patients' face to obtain a split-screen video-EEG documentation during the fMRI recording. Patients were asked to remain still during the scanning with eyes closed and do not fall asleep.

Functional data have been acquired using a Philips Intera system at 3 T and a gradient-echo echo-planar sequence from 30 axial contiguous slices (TR = 3,000 ms; in-plane matrix = 64 × 64; voxel size, 4 × 4 × 4) over one 10-min session (200 volumes) with continuous simultaneous EEG recording. A high-resolution T1-weighted anatomical image has been acquired to allow accurate anatomical localization of activations/deactivations. The volume consisted of 170 sagittal slices (TR = 9.9 ms; TE = 4.6 ms; in plane matrix = 256 × 256; voxel size = 1 × 1 × 1 mm).

### EEG Processing

BrainQuick System Plus software (Micromed) was used for offline correction of the gradient artifacts (24) and filtering of the EEG signal. In addition, the EEG data were exported in the .edf format and reviewed and analyzed by means of the BrainVision Analyzer 2.0 software (Brain Products, Munich, Germany). After removing the gradient and mean ballistocardiographic artifacts, an independent component analysis was performed on EEG data to isolate IEDs from physiological and artifactual activities.

Two experienced electroencephalographers reviewed the preprocessed EEG recordings independently (AEV, AR) to identify interictal epileptiform abnormalities (i.e., CTS) based on both spatial distribution and topography. When recognized, CTS were marked at peak. We classified patients as unilateral (right or left) in case of only one spike focus without migration; bilateral in the case that both foci were active. In this latter condition, left and right CTS were considered as independent in further analyses. The presence of sleep during fMRI recordings was checked by video recordings and by the presence of sleep spindles and K complexes.

### fMRI Data Preprocessing

The Matlab 7.1 and SPM12 (Wellcome Department of Imaging Neuroscience, London, UK) software was used for fMRI data preprocessing and analysis. All functional volumes were slice time corrected, realigned to the first volume acquired and smoothed with a 8 × 8 × 8 mm full width at half maximum Gaussian kernel. The six motion parameters derived from the fMRI preprocessing (translation and rotation in the *X, Y*, and *Z* direction, respectively) and a Volterra expansion of these (25) were used as covariates in the general linear model (GLM). Movement artifacts individuated by the analysis of EEG and video recordings (eye blink, deglutition, head movements, etc.) were considered as confounders in the model (26).

### EEG-fMRI Data Modeling

After preprocessing, for each patient, EEG and fMRI data were analyzed according to two different procedures:

- CTS were treated as single event and their onset exported in .mat file that describes the exact timing (in seconds) of CTS for fMRI time bin (TR = 3 s). The resulting timing files served as onsets for a GLM convolved with the standard hemodynamic response function (HRF) and its temporal derivatives (TDs). This analysis reflects the standard procedure generally adopted in previous works from our group and others (27–29) and will be named in the following paragraphs as “individual CTS.”
- Instead of treating CTS as single event, we computed the spike density, i.e., the number of ED for each time bin. As the aim was to use this information as regressor into the GLM model, we fixed the time bins equal to the TR, i.e., 3 s. Each spike density signal was then convolved with the standard HRF and its temporal first derivative (TD).

According to the EEG data analyses, two independent GLM models were created for each patient based on the effect of interest: “individual CTS” in model 1, “CTS density” in model 2. For both models, we specified as regressors of no interest the 24 realignment parameters (six scan realignment parameters from image preprocessing and a Volterra expansion of these) and the video based physiological facial movements. The resulting fMRI results [F-contrast or T-contrast as appropriate] were thresholded at *p* < 0.05, corrected for multiple comparisons [familywise error rate (FWE)] or at *p* < 0.001, uncorrected, if the subsequent BOLD maps did not reveal any changes at the more conservative threshold. In this latter case, an extent threshold of 10 contiguous voxels was applied to check for scattered BOLD changes. The statistical parametric maps were superimposed on the coregistered patients' anatomical MRI scans for localization purposes.

We choose not to merge the two regressors reflecting different CTS models in a common matrix as they would have been highly intercorrelated. We decided to employ two separate GLM to optimize the sensitivity of the first level analysis and the interpretation of the results (30).

#### Group-Level Analysis

Using the parameter estimates obtained by single-subject analyses, we performed two second level (group) random-effect analyses, one for each CTS model. To this end, the patients' realigned fMRI data were spatially normalized to a standard EPI template and smoothed again. A full factorial design was used, with hemodynamic shapes (HRF, TD) as factors. Subjects' age and gender were included in the model as covariates.

The threshold for statistical significance was set at *p* < 0.001 (uncorrected) and cluster extent of 10 voxels. The resulting statistical maps were displayed in MNI space and warped to the Population-Average, Landmark-, and Surface-based [PALS-B12 atlas in Caret (Caret, http://brainvis.wustl.edu/wiki/index.php/Caret:About; (31)].

Furthermore, we explored the differences by applying an exclusive masking procedure between the random analyses generated contrasts each related to the specific CTS model. In details, to isolate the brain regions that were significantly involved in the main effect “CTS density” but not in the main effect “individual CTS,” the contrast “CTS density > baseline” was exclusively masked by the contrast “individual CTS” and vice versa. SPM exclusive masks were thresholded at *p* < 0.05 uncorrected, whereas the contrasts to be masked were thresholded at *p* < 0.001. In this way, those voxels that reached a level of significance at *p* < 0.05 in the mask contrast were excluded from the analysis.

### Neuropsychological Assessment

General intelligence (IQ)—including verbal IQ (VIQ), performance IQ (PIQ), and full scale total IQ (TIQ)—was assessed using the Italian version of the Wechsler Intelligence Scale for Children (WISCIII and WISCIV). All scores were standardized for age and sex. For children with WISC III results, VIQ, PIQ, and TIQ where considered, whereas only WISC IV TIQ was analyzed.

### Clinical Correlation Analyses

We then further explored the potential relationship between CTS density BOLD changes and disease characteristics and neuropsychological scores in CECTS.

A whole-brain correlation analyses was used to test for a linear relation between BOLD signal changes relative to CTS (either individual event or density) with the disease features and neuropsychological scores. The following measures were considered: age at epilepsy onset, age at fMRI study, disease duration (in months), and neuropsychological parameters (verbal IQ, performance IQ, and full-scale total IQ). For this latter correlation, we limited the analysis to 16 CECTS being the neurophysiological evaluation available not for all patients (see below). The statistical significance level was set at *p* < 0.001 (uncorrected), with a cluster extent of 10 voxels.

## Results

### Clinical and Cognitive Findings

All the recruited patients completed the EEG-fMRI protocol. No subject's head motion exceeding 3 mm of translation or 3° of rotation. All EEG studies were recorded during resting quite wakefulness. No spindles and/or K complexes were observed. All patients except four (patients 2, 3, 8, and 19) demonstrated CTS during fMRI sessions. Of those, QI measures (TIQ, VIQ, PIQ) tests were available in 16 patients. The time lag between the neuropsychological tests and the fMRI experimental sessions ranged between 1 and 6.3 months. Disease's duration (in months) ranged between 0 and 103 months (mean, 24.8 months; median, 17.2 months). Table 1 summarizes the demographic and electroclinical data of the studied population. Neuropsychological data are reported in Table 2. The mean full-scale IQ was equal to 96.5 ± 14.6 (range, 71–124), mean PIQ = 100.88 ± 16.01 (range, 71–128), and mean VIQ = 99 ± 17.7 (range, 66–124). We did not observe any significant correlation between the cognitive measures and the total number of ED recorded during the fMRI experimental session (*p* = 0.063 Pearson's correlation), the ED density parameter (*p* = 0.065), as well as duration of epilepsy (*p* = 0.760) and age at seizures' onset (*p* = 0.864).

**Table 1:** Demographic and electroclinical data of CECTS.

**ID pt**.	**Disease's duration (mo)**	**Seizures type**	**AED**	**Spikes during fMRI (n)**
#1	1	FOS	Naive	P4 (560)
#2	5	FOS	Naive	–
#3	12	FOS	Naive	–
#4	2	FOS	Naive	F4 (250)
#5	30.8	FOS	Naive	T4 (90)
#6	0.15	FOSa	Naive	T4 (176) T3 (140)
#7	19.3	FOSa, GTCS	LEV	Pz P3 (67)
#8	32	FOS	OXC	–
#9	27.22	FOSa	LEV	T3, CP5 (511)
#10	7.28	FOS	Naive	T4 (92)
#11	0.16	FOS	Naive	T4 (641) F7 (525)
#12	32	FOSa	VPA	FC6 T4 (562) C3 (785)
#13	25.18	FOS	Naive	T4 (126)
#14	44.9	FOSa, GTCS	LEV	CP5 (52)
#15	9	FOSa	ETS + VPA	AF4 (26)
#16	3.9	FOSa	OXC	C4 (51)
#17	24.28	FOS	Naive	T4 (440) C3 (496)
#18	77	FOS	VPA	F8 (801) C3 (406)
#19	36	FOSa	VPA	–
#20	15.14	FOSa	CBZ	T4 P4 (693) Cz (279)
#21	12	FOS	Naive	T3 (84)
#22	1	FOS	Naive	C3 (185)
#23	11	FOS	Naive	C4 (47)
#24	45.9	FOSa, GTCS	Hydr + VPA + CBZ	P4 T6 (504)
#25	9.22	FOS	Naive	C4 (83)
#26	103	FOS	Naive	C4 (140)
#27	64	FOSa	VPA + LEV	C3 T3 (27)

*M, male; F, female; Y, yes; N, no; n, number; mo, months; FOS, focal onset seizures; FOSa, focal onset seizures with impaired awareness; GTCS, generalized tonic-clonic seizures; VPA, valproic acid; LEV, levetiracetam; OXC, oxcarbazepine; ESM, ethosuximide; Hydr, hydrocortisone; CBZ, carbamazepine. Spikes during fMRI sessions are described based on their topography and total number*.

**Table 2:** Neuropsychological measures of CECTS.

**ID pt**.	**TIQ**	**VIQ**	**PIQ**
#1	120	124	110
#4	97	97	97
#5	106	103	107
#6	81	89	77
#7	109	123	93
#9	110	91	109
#11	124	115	128
#14	101	110	128
#15	79	78	85
#16	104	101	106
#20	89	75	106
#23	84	77	93
#24	84	102	71
#25	100	108	92
#26	100	102	104
#27	75	66	95

*TIQ, full-scale total IQ; VIQ, verbal IQ; PIQ, performance IQ*.

### EEG During fMRI

A total number of 8,950 CTS (range between 27 and 801) were recognized, with a mean number of 255 CTS/patient and a mean density of CTS/30 s equal to 10,866 ± 11.46. Of those, 5,419 CTS mapped over the right hemisphere and 3,557 were left sided. CTS were classified as unilateral in 17 patients and bilateral in the remaining 6 cases (Table 1). Of those unilateral, 11 patients showed right CTS, while 6 left CTS. For each patient, the interictal events selected during scanning were similar to their routine EEG recordings; topography was checked for each patient and mapped over the centrotemporal and centroparietal leads in all cases (see Supplementary Figure 1).

### fMRI Findings

####  “Individual” CTS Analysis

**Figure 1:**
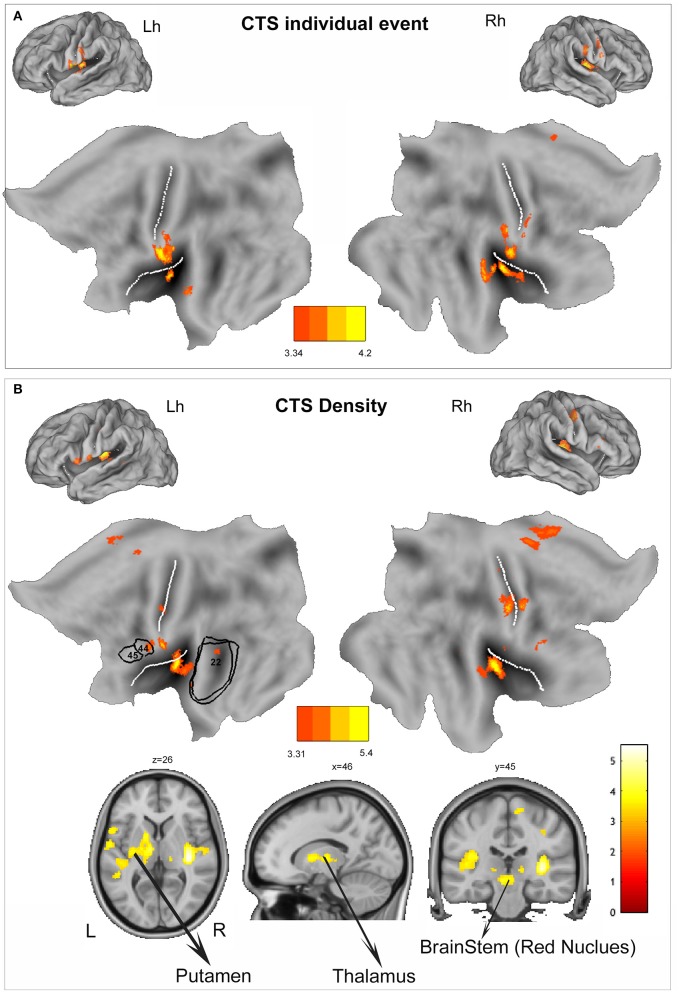
**(A)** Group-level individual centrotemporal spike (CTS) model (*p* < 0.001, 10 voxels extent). The functional maps are warped to the PALS-B12 atlas in caret (lateral view) for right (Rh) and left (Lh) hemisphere and to flat template. **(B)** Group-level density CTS model (*p* < 0.001, 10 voxels extent). The functional maps are warped to the PALS-B12 atlas in Caret (lateral view) for right (Rh) and left (Lh) hemisphere and to flat template. For localization purposes, functional results on the left hemisphere were plotted and compared against Brodmann areas of language areas (BA44, BA45, and BA22) indicated by the black numbers. In addition, to show the subcortical findings, BOLD changes have been overlaid into the canonical T1 0.5 mm image (coronal, axial, and sagittal slices) as implemented in FSL (FMRIB Software Library). L, left; R, right. The white lines on the PALS-B12 atlas and flat template show the central and sylvian fissure surface landmarks as implemented in Caret. The yellow-red color identifies positive BOLD changes. Negative BOLD changes were not observed. See text for details.

At group level, the regions that showed positive BOLD signal changes time locked to CTS are summarized in the Table 3 and Figure 1A. BOLD signal increases were observed at the bilateral postcentral gyrus (more on the right side) and bilateral insula. No decreases in BOLD signal were detected.

**Table 3:** Group level CTS-related BOLD findings.

	**Side**	**MNI coordinates**			***Z* score**
	**L/R**	***x***	***y***	***z***	
**Individual CTS**					
Insula-BA13	R	38	−16	8	4.65
Postcentral gyrus-BA3	R	64	−16	32	3.78
Insula-BA13	L	−52	−10	8	3.73
Postcentral gyrus-BA40	L	−54	−22	16	3.44
**CTS density**					
Insula-BA13^*^	R	36	−20	6	4.76
Precentral gyrus-BA4	R	54	−8	46	4.33
Insula-BA13	L	−38	−18	14	3.95
Superior temporal gyrus-BA22	L	−58	−4	8	3.60
Putamen	L	−32	−10	0	3.58
Inferior frontal gyrus-BA44	L	−50	14	2	3.49
Brain stem-red nucleus	L	−6	−24	−8	3.49
Cingulate gyrus-BA24	R	10	−2	44	3.49
Superior frontal gyrus-BA6	R	8	−14	66	3.49
Precentral gyrus-BA44	L	−60	10	8	3.45
Middle temporal gyrus-BA22	R	50	−42	6	3.30
Inferior frontal gyrus-BA45	R	54	26	12	3.20
Thalamus	L	−8	−14	−10	3.18
Thalamus	R	14	−24	0	3.14

*List of brain regions showing BOLD signal increases related to CTS either treated as single events either as density for time bin (p <0.001 uncorrected, 10 voxels extent threshold)*.

* *p < 0.05 corrected for FWE*.

*BA, Brodmann area; L, left; R, right*.

####  “CTS Density” Analysis

At single-subject level, we observed a good correlation between the BOLD response and the time course of the “density” regressor (see Supplementary Figure 2 for a representative example). CTS density random-effect analysis reveals the involvement of a more widespread cortico-subcortical network that encompasses the bilateral insula (BA13, global maxima over the right insular cortex), the bilateral sensory-motor cortex (BA4), more lateralized on the right side, the left inferior frontal gyrus (BA44), the right cingulate cortex (BA24), the right supplementary motor area (SMA) (BA6), the bilateral temporal cortex (BA22), the bilateral thalamus, and the putamen and red nucleus lateralized on the left side (Table 3, Figure 1B). No decreases in BOLD signal were detected.

#### CTS Density vs. Individual CTS

**Figure 2:**
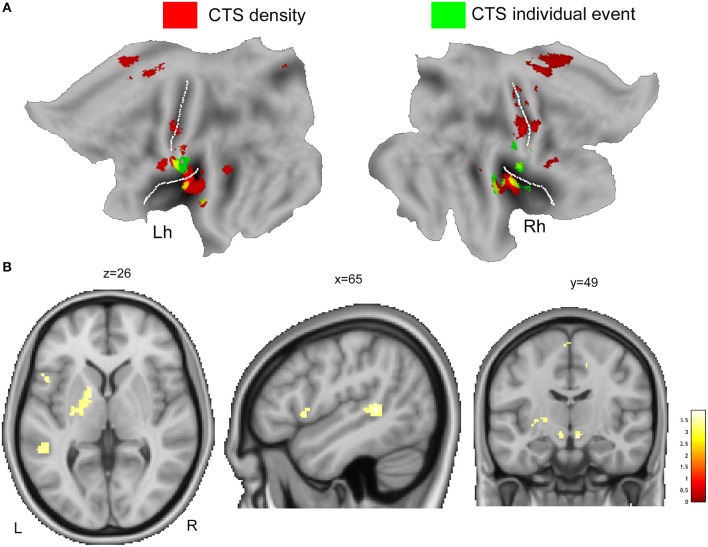
**(A)** The overlaid of individual CTS model (green color) and CTS density model (red color) is displayed onto the flat template as implemented by Caret for left (Lh) and right hemisphere (Rh). **(B)** The main effect contrast derived from group-level density CTS > baseline analysis was exclusively masked by the mask contrast “individual CTS > baseline,” at a threshold of *p* < 0.05, uncorrected for multiple comparison. See text for details. Clusters of activations are overlaid into the canonical T1 0.5-mm image (coronal, axial, and sagittal slices) as implemented in FSL (FMRIB Software Library). R, right; L, left.

Figure 2A displays the spatial overlap of the BOLD changes constrained to the two CTS models warped to PALS-B12 atlas, flat view. CTS density model reveals increased neuronal activity in the red nucleus, left putamen, left inferior frontal gyrus, left perisylvian cortex, and bilateral SMA, while CTS individual model does not (Figure 2B, Table 4). On the contrary, CTS single-event-exclusive BOLD correlates were observed at the bilateral (more right) sensory-motor cortex and right insula (Supplementary Figure 3).

**Table 4:** Exclusive masking findings CTS density vs. individual CTS model and vice versa.

	**Side**	**MNI coordinates**			***Z* score**
	**L/R**	***x***	***y***	***z***	
**CTS density** **>** **individual CTS**					
Brain stem, red nucleus	L	−6	−22	−8	3.77
Middle temporal gyrus-BA22	L	−52	−42	4	3.66
Putamen	L	−32	−10	0	3.58
Inferior frontal gyrus-BA47	L	−50	14	2	3.49
Medial frontal gyrus-BA6	R	12	−20	48	3.49
Medial frontal gyrus-BA6	L	−2	−12	66	3.23
**Individual CTS** **>** **CTS density**					
Postcentral gyrus-BA3	R	60	−18	32	3.91
Insula-BA13	R	46	−4	10	3.60
Precentral gyrus-BA4	L	−60	−14	38	3.49

*List of brain regions showing BOLD signal increases related to the CTS density vs. individual CTS model and vice versa (p < 0.001 uncorrected, 10 voxels extent threshold)*.

*BA, Brodmann area; L, left; R, right*.

#### Correlations Between BOLD Signal and Clinical Measures

**Figure 3:**
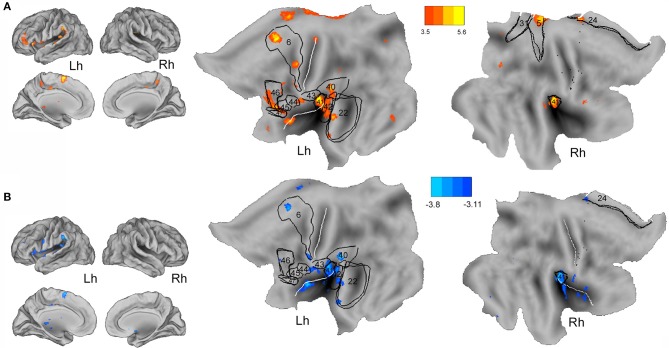
Whole-brain correlation analysis between CTS density-related BOLD changes and disease's duration **(A)** and age at fMRI **(B)** are warped to the PALS-B12 atlas in caret (lateral and mesial view) for right (Rh) and left (Lh) hemisphere and to flat template. See text for details. For localization purposes, functional results were plotted and compared against Brodmann areas indicated by the black numbers. The white lines on the PALS-B12 atlas and flat template show the central and sylvian fissure surface landmarks as implemented in Caret. The yellow-red color identifies the positive correlations, the light-blue color the negative correlations.

Whole-brain correlation analyses using individual clinical characteristics of the CECTS patients disclosed a linear positive relationship between the interictal discharge-related BOLD changes (CTS density model) and disease duration at the bilateral insula (BA13), bilateral cingulate cortex (BA31, BA24), bilateral auditory cortex (BA41–42), the left supramarginal gyrus (BA40), left middle temporal gyrus (BA22), left dorsolateral prefrontal cortex (BA46), left inferior frontal gyrus (opercular and triangular part) (BA44–45), and left superior frontal gyrus (BA6). Interestingly, these BOLD changes survive at a more conservative threshold of *p* < 0.05 corrected for FWE (Figure 3A). A similar hemodynamic map was obtained by correlating CTS density BOLD changes with patients' age (at fMRI study) although of the opposite sign: a negative relationship was indeed observed at the bilateral insula, bilateral cingulate cortex (BA24), bilateral auditory cortex (BA41–42), left supramarginal gyrus (BA40), left middle temporal gyrus (BA22), left inferior frontal gyrus (BA44), and left superior frontal gyrus (BA6) (Figure 3B). In other words, the younger the patient and the longer the disease, the higher was the metabolic gain of the perisylvian and the language circuitry of the brain in case of very frequent CTS. Intriguingly, the individual event CTS analyses did not reveal any linear correlation with patients' age, while a positive relationship was detected between the disease's duration and CTS-related BOLD map at the right posterior cingulate cortex and right precuneus (data not shown). Correlation between CTS BOLD changes and neuropsychological measures as well as CTS BOLD changes and age at epilepsy onset did not revealed any significant relationship for both the specified GLM models.

## Discussion

The present study is innovative for different aspects. First, we explore the hemodynamic counterpart of the ED density measure during wakefulness in a cohort of patients affected by CECTS. Using EEG-fMRI, instead of individual events, we modeled the number of ED for time bin. The resulted continuous regressor expresses the frequency (i.e., density) of ED for time window along the entire fMRI session at single-subject level. The rationale behind this approach is not trivial as abundance of ED is recognized as a prognostic factor of the neurocognitive outcome in CECTS (16, 31) and often influences the clinicians' decision tree including treatment (32, 33). Second, our analyses provide a significant contribution within the “puzzle” of evidences that try to explain the complex relationship between epilepsy and cognition in CECTS. With respect to the individual spike analysis, the ED density model BOLD maps reveal the engagement of brain hubs beyond the epileptogenic zone demonstrating how individual ED affects a limited territory, but at the same time, their number can influence the activity of a broader network, comprising nodes of relevance for cognitive functions, and in particular for language. This effect appears to be greater in youngest patients and in those with longer disease's duration, thus confirming the hypothesis of an age-dependence effect of ED on the cognitive development (34) and further supporting the necessity of an early and patient's tailored neuropsychological assessment in CECTS, especially in case of high frequency, even diurnal, ED (34, 35).

### The ED Density Effect on Cognition in CECTS

The importance of ED density parameter and its impact on cognition have been largely discussed in relation to the non-rapid eye movement sleep ED activation, phenomenon described in different epileptic conditions of the same spectrum that includes CECTS as the mildest extreme (36). It has been argued that the negative effect of epileptic discharges during sleep might reflect the impairment of the physiological sleep-related synaptic homeostasis processes (37, 38) that, if occurring in the critical period of development, may disrupt cognitive functions and behavior, hence interfering with the learning process occurring in wakefulness (17). By converse, the effect of diurnal ED density has been poorly investigated, especially in CECTS, and to our knowledge, the present report is the first study that addresses this issue specifically using a functional neuroimaging protocol. Previous clinical studies support the existence of a relationship between the number of spikes on awake EEG and neuropsychological performances. Although in a limited number of patients, the visual discrimination between words and pseudo-words in a reading test has been confirmed to be impaired in patients with frequent diurnal CTS (19). Children affected by different epilepsy syndromes (including CECTS) with diurnal ED in ≥10% of the EEG record showed impaired central information processing speed, short-term verbal memory, and visual-motor integration (39). This effect was seen independently from other EEG-related and epilepsy-related characteristics and from epilepsy syndrome diagnosis (39). Previous EEG-fMRI and magnetoencephalography-fMRI studies demonstrated that diurnal ED in focal childhood epilepsies (including CECTS) may affect the oscillatory synchrony, and the organization of spontaneous network connectivity in the developing brain and changes in the network topology might account for cognitive impairments (13, 15). Interestingly, children with less resilient (or highly vulnerable) networks were found to be prone to a greater frequency of ED and that the combined contribution of network changes and ED is strongly associated with IQ (13). Functional neuroimaging studies in epileptic encephalopathies including electrical status epilepticus during sleep (ESES), a condition characterized by an elevated amount of ED, demonstrated a reliable pattern of network activation including the perisylvian region, temporal, parietal, and cingulate cortex. In addition, besides the network related to epileptiform discharges, there are changes in brain regions pertaining to of the default mode network (28, 40, 41). This latter finding was explained as a remote diurnal ED effect able to explain cognitive deficits in patients with ESES (41). The results of our current report confirm and expand these previous observations. Either the ED density and the ED individual models revealed the involvement of the pericentral and the perisylvian regions, particularly of insula (Figure 1). Both these areas can be regarded as key zones for the CTS generation (42) and similar EEG-fMRI findings in CECTS (15) and even in other conditions of the spectrum (ESES, Lennox-Gastaut) (28, 43) point in this direction. A recent ictal source imaging study in CECTS showed the activation of the operculo-insular area time locked to the contralateral focal myoclonic jerks, emphasizing the role of this network for seizures generation (44). Nevertheless, it has been argued that the insular and, in general, the perisylvian involvement reflects the propagation of ED, and they might contribute to cause specific neuropsychological deficits (28). The ED density model, but not the ED individual model demonstrated a diffuse cortical frontal and temporal activations including the right anterior cingulate cortex, the bilateral SMA, and the anterior and posterior speech cortex [the left inferior frontal gyrus (Broca's area) and the left superior temporal gyrus (Wernicke's area)] (see Figure 1B). Even the SMA involvement can be linked to a functional disruption and hence reorganization of the language network (45). A direct effect of CTS on the language-related areas has been largely documented either as a transient disturb (15, 46, 47) and more long-lasting morphological changes (48–50). As far as the anterior cingulate (ACC) involvement, it probably reflects attentional difficulties as frequently documented in CECTS even at onset and free of medication (35). Interestingly, altered cortical thickness in CECTS patients with comorbid attention-deficit/hyperactivity disorder involved the cingulate gyri (51). Beside cortical involvement, ED density measure was associated with increase in BOLD signal of putamen, thalamus, and red nucleus. The putamen is of particular interest given the growing evidence for its selective anomaly in CECTS (14, 52). Previous findings observed an increase variability (calculated based on functional connectivity measure) in the striatal (dorsal putamen)–sensorimotor circuits during CTS, and this excessive variability was related to highly frequent ED (14). Our results point in the same direction and support the hypothesis that the dynamic characteristics of interictal epileptic activity act as modulator of the oscillatory dynamics in the striatal–sensorimotor epileptogenic circuitry. Interestingly, the left putamen and motor cortex have been associated with the initiation and execution of overt relative to covert speech (53). Taken together, the BOLD map revealed by the density model provide further information within the complex interaction between the (diurnal) epileptic activity and the brain functionality, highlighting the involvement of core node (putamen, SMA, anterior cingulate cortex) and networks (language, attentional) likely interfering on the cognitive profile of these children.

### The Age-Dependence Effect of ED Density on Normal Neurodevelopment

We demonstrated a linear relationship between the BOLD ED density-related changes and both the patients' age (negative correlation) and disease duration (positive correlation). In details, the longer the disease and the younger the patient, the higher is the engagement of the bilateral perisylvian cortex (insula) and a complex network of brain hubs pertaining to the language processing stream (Figure 3). In details, ED density measure interferes on regions responsible for the verbal fluency (Broca's area) (53), speech comprehension (Wernicke's area) (54), phonological retrieval and articulatory words processing (supramarginal gyrus) (53, 54), and auditory speech processing (Heschl's gyrus) (53). Even the cingulate and the insular cortex involvement could be regarded in the contest of their participation in words production, especially articulatory planning (insula) and lexical decision (anterior cingulate cortex) (53). Of interest, the individual spike model did not end with similar results, but rather, it shown a positive relationship between the ED-related metabolic activity of the posterior default mode network (precuneus and posterior cingulate cortex) and the disease's duration variable. CECTS is an age-dependent epileptic condition and is therefore clear that maturational factors are important in the development and expression of the disease (34, 55). Recurrent epileptic activity in critical period of life would likely influence and interfere with brain development, aided by the greater neuroplasticity and less functionally specialized neural networks (56). In addition, perisylvian, prefrontal, and cingulate cortices undergo a long developmental process and are sensitive to environmental influences and intrinsic physiological perturbations, as ED, throughout childhood and adolescence (57, 58). GRIN2A knocked out mice, a genetic model of epilepsy-aphasia spectrum (encompassing CECTS, Landau–Kleffner syndrome, and ESES), displayed impaired vocal communication as well as microstructural diffuse brain alteration in a specific development time window, corresponding to the human school-age/pre-adolescence (59, 60). In CECTS, morphometric analyses revealed diffuse increases in gray matter volumes that inversely correlated with age (49). In addition, the rate of physiological changes in cortical thickness during development was higher in CECTS than controls, and the time to reach normative values was delayed (49, 50, 61). This raise the possibility that the natural course of CECTS may reflect a deviation from the normal developmental trajectory in core regions during critical periods of life. However, such evidences did not consider specifically the effect of ED on causing these age-related disruptions.

In the current report, we shown that the BOLD effect of ED density is negatively correlated with age; on parallel, this effect increases over time given the linear positive correlation with the disease's duration. We can thus hypothesize that an elevated ED density more than the spike itself might alter specific cortical functionality during the critical period for language acquisition and consolidation (62). Persisting language problems following remission might also depend on the long-lasting disrupted effect of frequent epileptic activity on brain circuits during critical epochs of development and specialization. Unfortunately, selective language evaluation by specific subtests is lacking in the CECTS cohort examined, representing a limitation of the current study, and make our assumptions speculative. Nevertheless, the present findings are noteworthy, as they lay the groundwork for additional important future studies. We did not observe a correlation between the ED density (and even individual spike) BOLD changes and the IQ variables. The existence of a variable time lag between the cognitive evaluation and the fMRI experimental sessions across our CECTS patients might account for this negative finding. Nevertheless, such lack of correlation might corroborate the previous suggestion regard the IQ as low sensitive measures for the cognitive assessment in these patients (14). It is commonly reported that children with CECTS display normal-range IQ on a background of specific cognitive difficulties (34, 35). In addition, it raises the issue of the need for appropriate neuropsychological testing, individually tailored to specific deficits and interpreted in the light of the neurophysiology and functional neuroimaging data (63, 64).

Translated to the clinical practice, our findings suggest that CECTS patients with high-density EEG abnormalities during wakefulness need a comprehensive neuropsychological assessment including especially attentional skills and language abilities. In addition, these patients may be considered for specific neuropsychological or pharmacological treatment (if indicated based on several clinical and EEG parameters) early in their clinical history, and the disappearing of ED or a reduction in their frequency might be considered a prognostic factor for a better neuro-behavioral outcome.

### Methodological Considerations

Previous EEG-fMRI studies in patients (adults and children) with focal epilepsies and frequent interictal spikes on EEG (range between number of spikes >100/35 min to >200/20 min) argued about the validity of GLM at such high spiking rate and suggested different statistical approaches that assume nonlinearity of the BOLD response (65, 66). To note, these studies were performed in patients with different epileptic conditions rather than CECTS. Previous EEG-fMRI evidence in CECTS patients, even in case with frequent ED, were performed assuming the validity of the GLM and demonstrated highly reproducible and stable findings (15, 20, 29, 67–69). Even in patients with continuous spike and wave during sleep (with more than 1,000 spikes/20 min for subject), and other self-limited focal epilepsies, the GLM was adopted (27, 28). Supplementary Figure 2 shows a good correspondence between periods of increased spiking rate and BOLD amplitude. Based on these evidences and also the need to get comparable results with previous findings, we assumed the GLM to be valid despite the high number of spikes. To note, as for ESES spikes in CECTS do not occur with temporal regularity.

### Study Limitations

We are aware that the present work is limited in several ways. First, it is limited by the small sample size, especially in relation to the subsample of patients with available cognitive assessment. Second, antiepileptic medications (AEDs) might have confounded BOLD findings, by altering the excitability and the neurovascular coupling. AED heterogeneity across the CECTS population prevented to test for their specific effect on the ED density-related BOLD maps. However, when we compared the CTS density BOLD maps in patients naive (*N* = 13) vs. patients on AED (*N* = 10), we did not observe any significant difference, even at low threshold. In addition, ED measures were not statistically different in patients naive compared to patients on AEDs (one sample *t* test, *p* = 0.44). Cognitive tests' results can be influenced by AEDs, especially in case of polypharmacy. Nevertheless, no patient was taking AEDs that are well-known to exert adverse effects on cognition, such as barbiturates, benzodiazepines, and topiramate.

Third, our cohort was not homogenous as patients were mostly at different stages of their disease at the moment of fMRI and intellectual testing, and, for those available, the time window between fMRI and neuropsychology was different across them. Finally, but probably more important, the neuropsychological assessment was limited to the IQ measures and our data lack of comparison with normal healthy peers (70).

## Conclusion

There is mounting evidence that CECTS is a neurodevelopmental disorder with key neurocognitive impairments in speech, language, attention, and executive and motor functions (34).

It is generally accepted that neurobehavioral functioning in CECTS is multifactorially determined (71), with epileptiform activity “per se” being one of the main responsible for documented neuropsychological and behavioral problems (13, 15). In CECTS, more than other conditions like ESES is clear that the total amount of ED alone is not sufficient to predict the clinical course. Our work provides additional knowledge in this contest and highlights the importance of the ED frequency, even during wakefulness, as a prognostic factor to be taken into account during the diagnostic and therapeutic workup of CECTS patients. Of note, a timely evaluation of diurnal ED frequency is of importance, as it appears to increase its impact on normal brain functioning over time. The ED density parameter, together with the conventional clinical and neuropsychological assessment, might represent an additional feature useful to determine the severity of epilepsy and could help an early decision about whether to start AED treatment.

## Data Availability Statement

The datasets generated for this study are available on request to the corresponding author.

## Ethics Statement

The studies involving human participants were reviewed and approved by Comitato Etico dell'Area Vasta Emilia Nord, Via Largo del Pozzo 71, 41124, Modena. Written informed consent to participate in this study was provided by the participants' legal guardian/next of kin.

## Author Contributions

AEV, PA, GC, and SM outlined the subject of the research theme and interpreted the literature and wrote the manuscript. AEV, and SM obtained ethical permission to perform the research and agreed to be accountable for all aspects of the work. AEV, GC, MF, EC, PB, AG, AV, MC, MS, PV, GGo, GGe, SM, BP, FP, and BD searched the patient files and collected the original data. AEV, PA, AR, and FT analyzed the data.

### Conflict of Interest

The authors declare that the research was conducted in the absence of any commercial or financial relationships that could be construed as a potential conflict of interest.
