# Supplementary material for: Mapping the Effect of Interictal Epileptic Activity Density During Wakefulness on Brain Functioning in Focal Childhood Epilepsies With Centrotemporal Spikes
Source: Front Neurol. 2019 Dec 19;10:1316. doi: 10.3389/fneur.2019.01316 (PMC6930928; doi:10.3389/fneur.2019.01316)
Supplement: Supplementary file 1 [file Data_Sheet_1.pdf]

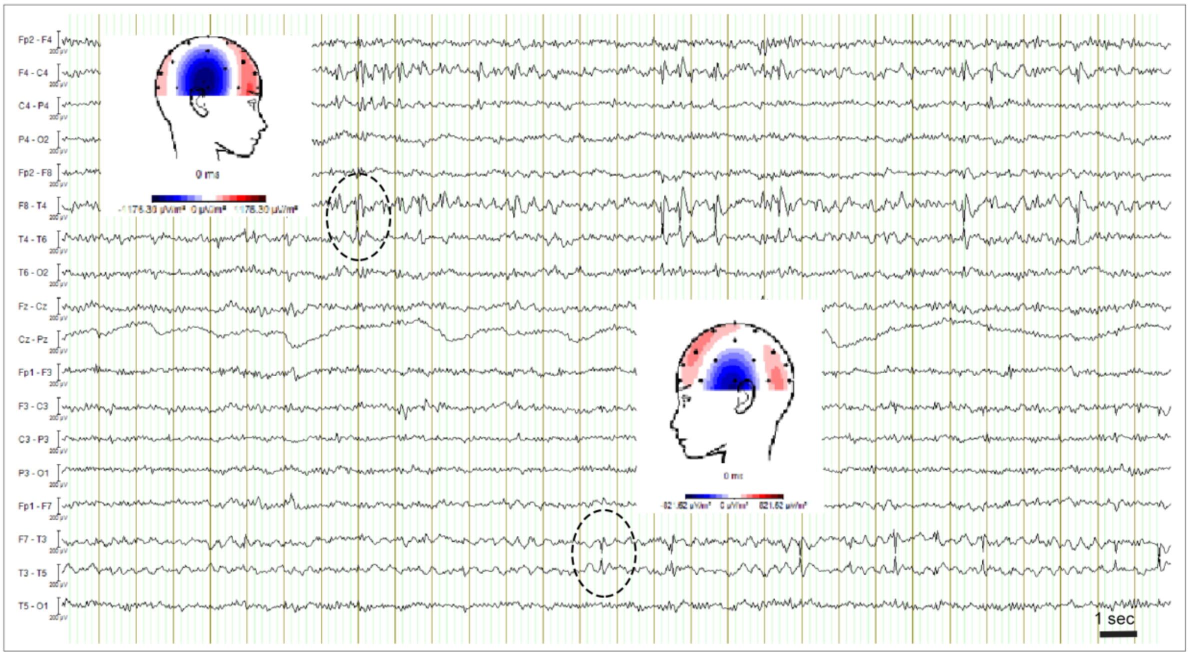

Supplementary Figure 1. Representative EEG page acquired during fMRI scanning in a patient with CECTS (Pt#6). The EEG trace is displayed in bipolar montage and shows clear bilateral and asynchronous CTS over the frontal-temporal leads with related topography estimated at the peak of the averaged spikes. CTS are highlighted by black ellipses. Sec: second.

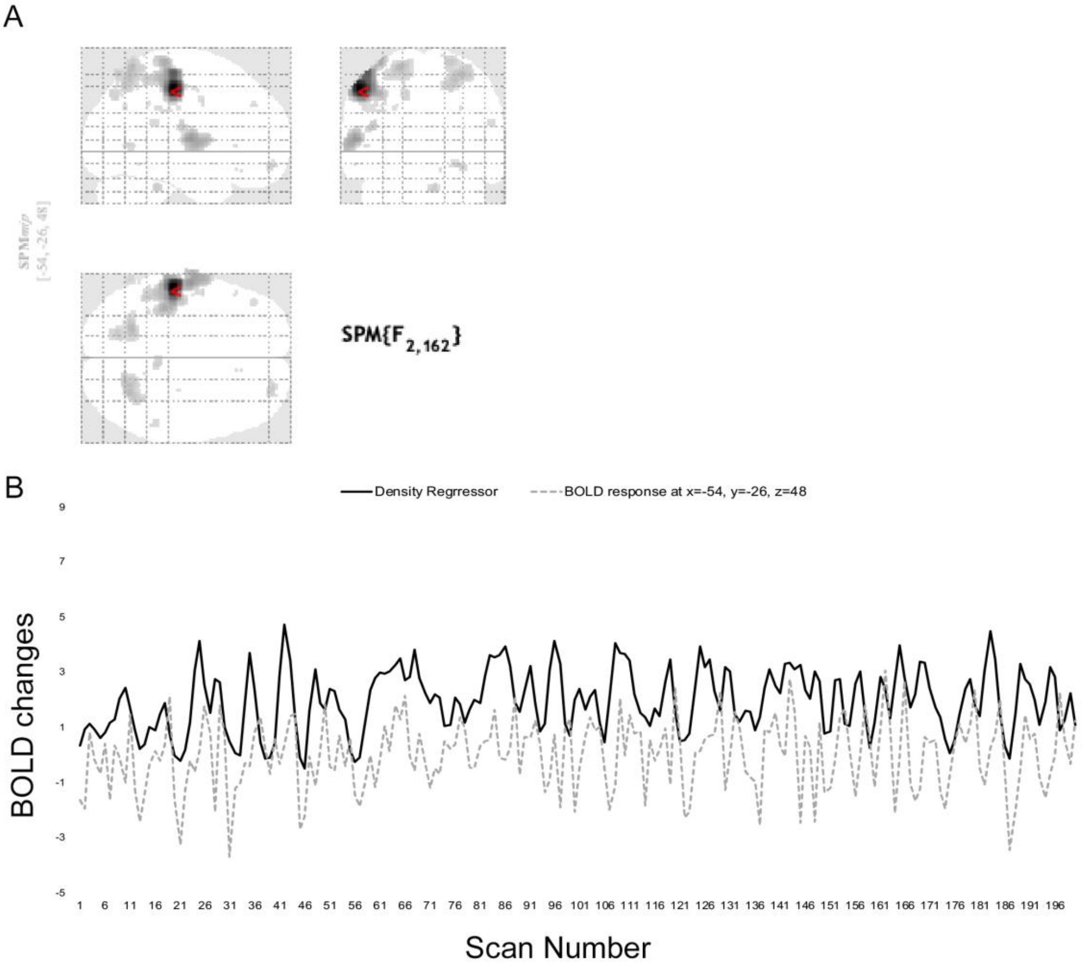

Supplementary Figure 2. Panel A: BOLD response that was obtained for Patient #12 (left CTS, total number: 785) in a voxel with the maximal f-value in left postcentral gyrus. The bottom graph (Panel B) shows the relation between variation of the fitted positive BOLD response and spike density. The x-axis displays the scan number (between 0-200). Note that frequent spikes were associated with an increase in BOLD response and gaps between spikes corresponded well with BOLD decreases.

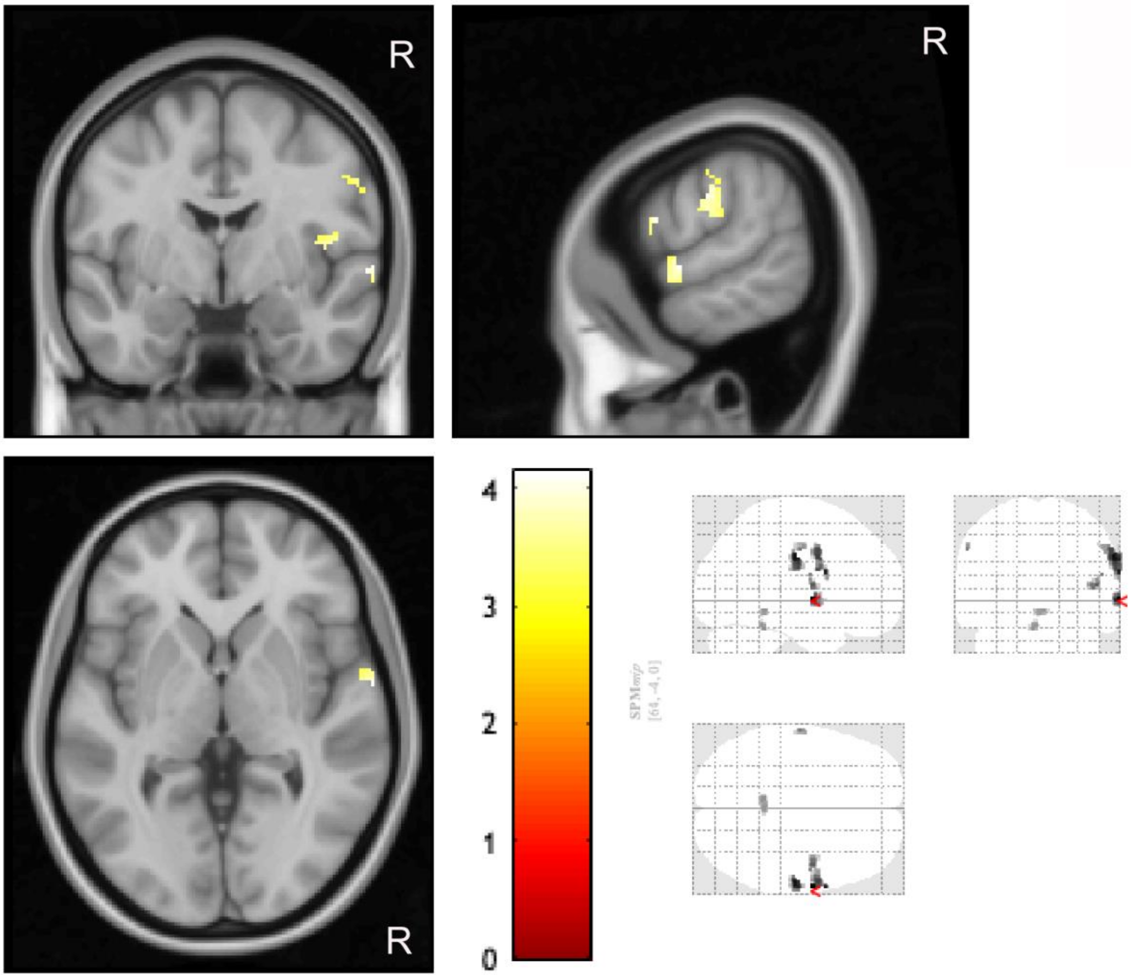

Supplementary Figure 3. The main effect contrast derived from group-level individual CTS>baseline analysis was exclusively masked by the mask contrast "CTS density>baseline", at a threshold of $p < 0.05$, uncorrected for multiple comparison. See text for details. Clusters of activations are overlaid into the canonical T1 0.5 mm image as implemented in FSL (FMRIB Software Library). R: Right.
